# Low transthyretin concentration linked to adverse prognosis in elderly inpatients

**DOI:** 10.1186/s12877-024-05467-3

**Published:** 2024-10-30

**Authors:** Ting Wang, Zhi-kai Yang, Yu-hao Wan, Ke Chai, Ying-ying Li, Yao Luo, Min Zeng, Ning Sun, Song Zou, Hua Wang

**Affiliations:** Department of Cardiology, Beijing Hospital, National Center of Gerontology, Institute of Geriatric Medicine, Chinese Academy of Medical Sciences, Beijing, P. R. China

**Keywords:** Transthyretin, Prognosis, Elderly inpatients

## Abstract

**Background:**

To investigate the association between low transthyretin (prealbumin) concentration and mortality or readmission for all causes in elderly inpatients.

**Methods:**

This analysis is based on a prospective cohort study conducted from September 2018 to April 2019 in ten wards of three tertiary referral hospitals in Beijing. Patients aged 65 years or older were enrolled, and their clinical data, laboratory test results, and auxiliary test results for patients were collected. A three-year follow-up was conducted with patients. Based on the 5th and 95th percentiles of transthyretin concentration, patients were split into three groups. The correlation between transthyretin concentration and the outcome of elderly hospitalized patients was investigated. The primary outcome of the research was death or readmission from all causes within three years.

**Results:**

Among the 636 individuals in the study, 335 (52.7%) were males, with a median age of 74.7 years (interquartile range [IQR]: 69.3–80.1). During a median follow-up period of 1,099.0 days (IQR: 1,016.3-1,135.0), 363 individuals (57.0%) experienced all-cause mortality or readmission events. Patients with transthyretin concentrations at or below the 5th percentile had a significantly increased risk of all-cause mortality or readmission compared to those with concentrations between the 5th and 95th percentiles (hazard ratio [HR]: 2.25; 95% confidence interval [CI]: 1.55–3.26). Even after adjusting for potential confounders, low transthyretin concentration remained an independent risk factor for poor prognosis in elderly inpatients (HR: 1.84; 95% CI: 1.03–3.28). Since women have consistently lower baseline transthyretin levels than men, we performed gender-specific analysis. We found that low transthyretin concentration is an independent risk factor for adverse prognosis in elderly male inpatients (HR: 2.99; 95% CI: 1.35–6.62) but not in females.

**Conclusions:**

Low transthyretin concentrations are associated with increased all-cause mortality or readmission in elderly inpatients, particularly among male patients.

**Graphical Abstract:**

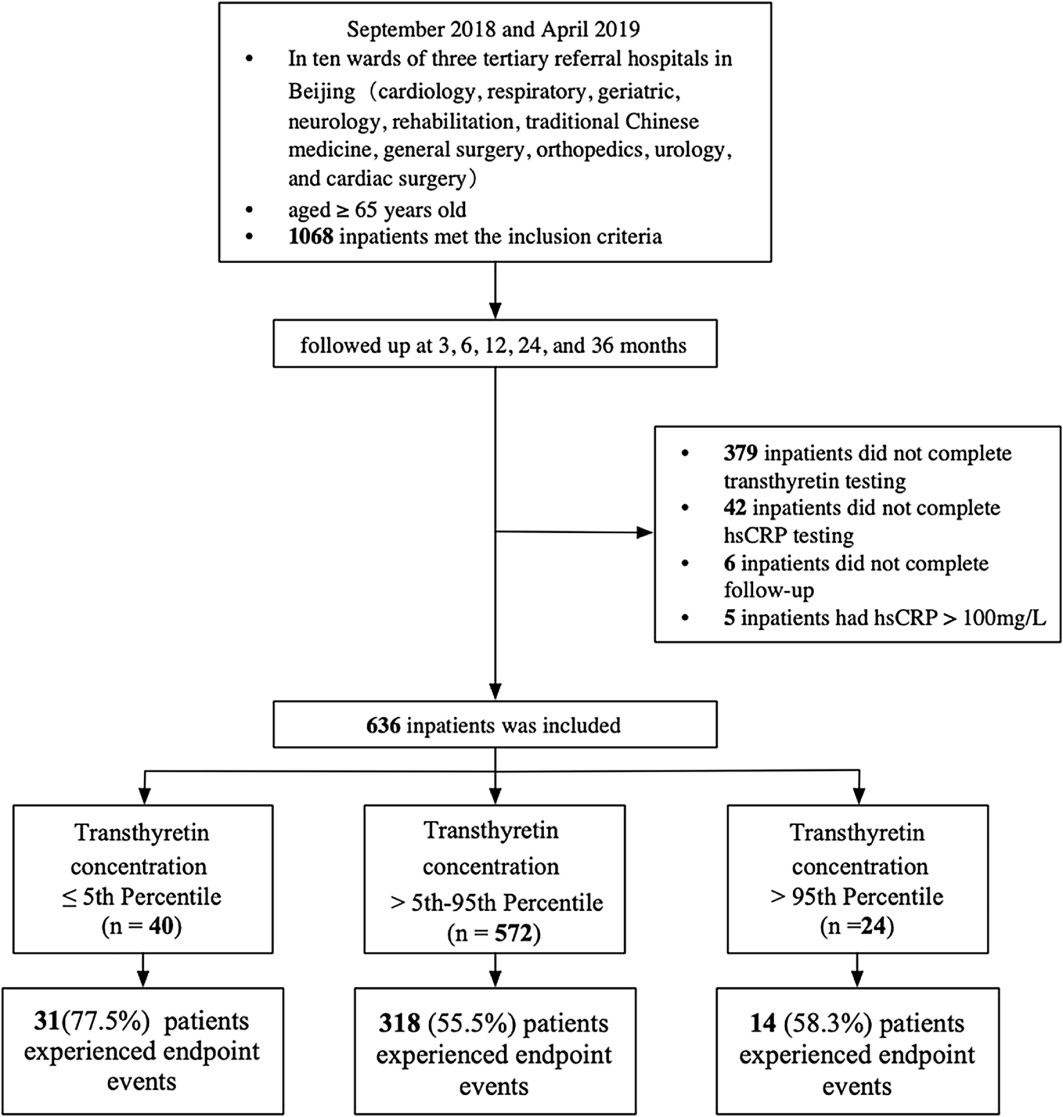

**Supplementary Information:**

The online version contains supplementary material available at 10.1186/s12877-024-05467-3.

## Background

China is currently facing a significant demographic challenge as it has a rapidly aging population with a sizeable number of geriatrics. According to the results of China’s seventh national population census, individuals aged 60 and above accounted for 18.7% of the total population, with 190.64 million individuals aged 65 and over representing 13.5% of the total population [1]. According to the China Health Statistics Yearbook, the hospitalization rate of residents aged 65 years and above has been increasing steadily, from 8.4% in 2003, 15.3% in 2008, and 19.9% in 2013 to 27.2% in 2018 [2, 3]. Given this trend, predicting the prognosis of elderly inpatients as early as possible and developing individualized treatment plans based on the specific conditions are crucial for improving the prognosis and the quality of life of elderly inpatients.

Transthyretin, also referred to as prealbumin, is a 127-amino acid protein produced primarily by the liver [4]. In vivo, transthyretin forms a stable tetramer, under normal physiological conditions [5]. Due to its small molecular weight, short half-life, and high reactivity to protein status, transthyretin is considered an indicator of nutritional status [6, 7]. Additionally, transthyretin is an inverse acute phase reactant, and is also frequently used as a clinical indicator of inflammation [6, 7]. Under the effect of gene mutation or aging, the tetrameric structure can dissociate into monomers and misfold into amyloid fibers, which can be deposited in the heart, nerve, kidney, eye, and other parts, causing damage to various systems [8].

Several studies have demonstrated that transthyretin has significant predictive value for morbidity and mortality in some patients, including those undergoing surgery for colorectal cancer [9], cardiac surgery [10], radical gastrectomy [11], stroke [12], systemic sclerosis outpatients [13], heart failure [14]and others. Despite these findings, to our knowledge, no studies have explored the relationship between low transthyretin concentrations and prognosis in elderly hospitalized patients across different wards. Therefore, this study aims to evaluate the correlation between low transthyretin concentrations and prognosis in elderly inpatients.

## Methods

### Study design and participants

This study is based on a previously conducted prospective observational cohort study of elderly inpatients. From September 2018 to April 2019, the cohort study screened a total of 1,068 elderly inpatients who were admitted to ten wards, encompassing the internal medicine and surgical wards, in three tertiary referral hospitals in Beijing, China [15]. Only inpatients aged 65 years and above were considered eligible for the study, and those who refused to provide informed consent or were unable to cooperate with the assessment were excluded. The follow-up period spanned three years, during which major adverse events were recorded at three, six, twelve, twenty-four, and thirty-six months following enrollment. The study excluded 379 patients who did not complete transthyretin testing, 42 who did not complete high-sensitivity C-reactive protein (hsCRP) testing, 6 who were lost to follow-up, and 5 who had hsCRP > 100 mg/L (since transthyretin is an inverse acute-phase reactant), resulting in the final inclusion of 636 patients. The 636 patients were recruited from Beijing Hospital and Beijing Tsinghua Changgung Hospital. Comparison of baseline characteristics between patients with and without transthyretin measurements is presented in Supplementary Table 1.

### Transthyretin concentration and other covariates

#### Transthyretin concentration

In this study, serum samples were used to measure the concentration of transthyretin using an immunoturbidimetric assay. Beijing Hospital utilized the Beckman AU5800 biochemical analyzers (Beckman, USA) with reagents from DiaSys, and Beijing Tsinghua Changgung Hospital used the Siemens ADVIA 2400 biochemical analyzers (Siemens, USA) with Siemens ADVIA reagents. Multivariate linear regression analysis of transthyretin concentrations showed that the hospital variable had no significant effect on transthyretin levels (*P* = 0.267), indicating that the different measurement methods used by the hospitals did not affect the consistency of the transthyretin measurements.

#### Covariates

The study’s covariates were selected in accordance with present literature showing relevance to transthyretin concentration. The covariates included age, sex, nutritional status and lifestyle factors (body mass index [BMI], albumin, cholesterol, triglycerides, low-density lipoprotein, diabetes, smoking, alcohol intake, physical inactivity), liver synthesis capacity and liver cell damage (albumin and alanine aminotransferase [ALT]), kidney function (creatinine), and inflammation (hsCRP) [16]. Additionally, we collected the patient’s frailty status and cardiovascular disease. We used the Research Electronic Data Capture (REDCap) to manage the study data.

Age, sex, BMI, albumin, cholesterol, low-density lipoprotein, triglyceride, ALT, creatinine, and hsCRP were measured at admission. Diabetes, smoking, and alcohol intake were self-reported by the patients and categorized into two groups: never and others. Physical activity was defined as walking 2.5 h (men) or 2 h (women) per week. We used the Fried phenotype, which consists of five criteria (unintentional weight loss, exhaustion, low grip strength, slow walking speed, and slow physical activity), to assess frailty. Patients with three or more characteristics were diagnosed with frailty, while those with one or two characteristics were classified as prefrail, and those with no characteristics were considered robust [17]. Previous cardiovascular disease was obtained from patient self-report and medical record review.

### Primary outcome

The primary endpoint was death or readmission from all causes within three years. Patients were followed up at three, six, twelve, twenty-four, and thirty-six months, either in the clinic or by telephone, to record major adverse events.

### Statistical analysis

The data analysis was conducted using R version 4.2.0. Shapiro-Wilk tests and quantile-quantile plots were used to determine the normal distribution of continuous variables. Continuous variables that followed a normal distribution were reported as mean ± standard deviation, whereas non-normally distributed continuous variables were presented as median (interquartile range [IQR]: 25th to 75th percentiles). Categorical variables were expressed as percentages. For trend analysis across transthyretin percentile groups, we employed the Jonckheere-Terpstra-Kendall trend test. Survival curves were constructed using the Kaplan-Meier method, and survival time were compared using the Log-rank test. Univariate Cox regression analysis was initially conducted to identify relevant variables. Variables included in the multivariate Cox regression analysis were those associated with the outcome (mortality and readmission) as well as those related to transthyretin concentration. The multivariate Cox proportional hazards regression model was utilized to determine risk factors of endpoint events, with hazard ratios (HR) and 95% confidence intervals (CI) reported. Statistical significance was considered when *P* < 0.05.

## Results

A total of 636 elderly inpatients were enrolled, including 335 males (52.7%). The median (IQR) age of the participants was 74.7 (69.3, 80.1) years, and the transthyretin concentration was 24.00 (20.00, 28.00) mg/dL. To evaluate the relationship between transthyretin concentration and prognosis in this population, patients were classified into three groups in accordance with their transthyretin concentration: those at or below the 5th percentile, those within the 5th to 95th percentile range (control group), and those greater than the 95th percentile.

### Description of baseline group characteristics

**Fig. 1:**
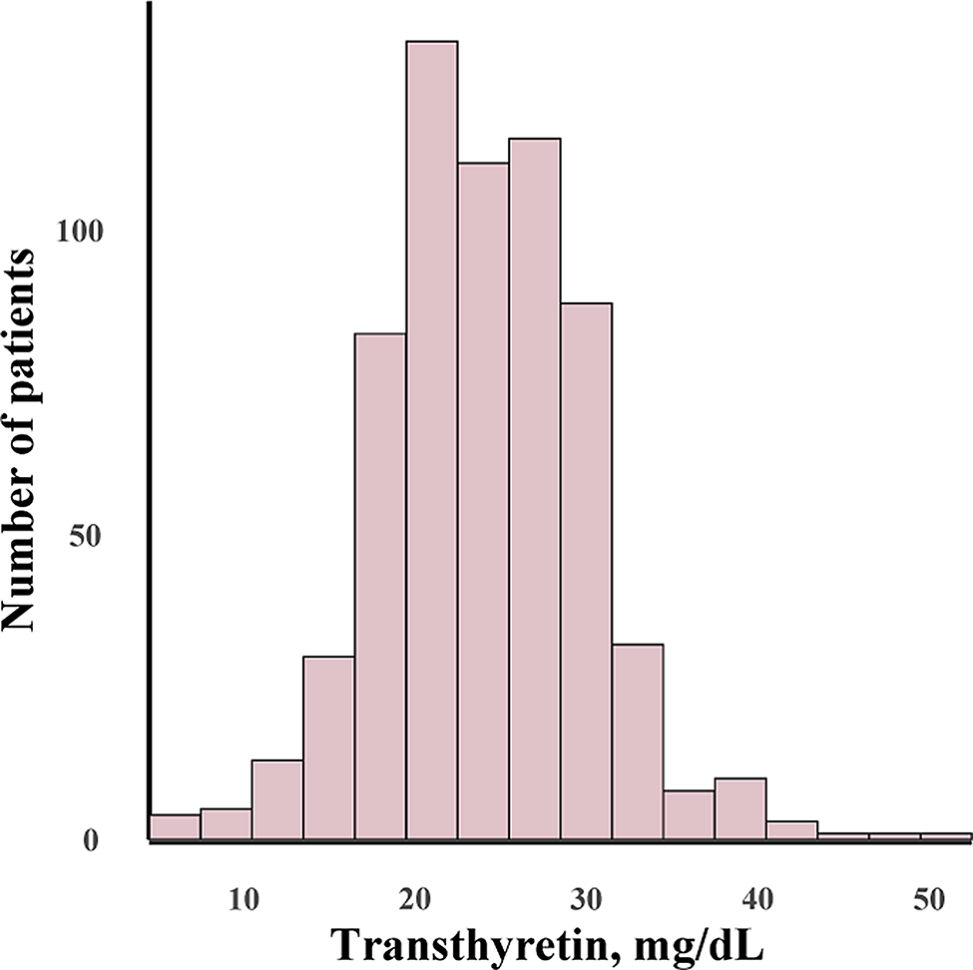
Distribution of transthyretin concentrations in the study population

The transthyretin concentrations at the 5th and 95th percentiles were 15.00 mg/dL and 34.00 mg/dL, respectively, and the patients were categorized into three groups: [4.59,15.00], (15.00,34.00], (34.00,51.00]. The distribution of transthyretin concentrations is illustrated in Fig. 1. As shown in Table 1, age, BMI, diabetes, alcohol intake, physical activity, frailty, albumin, triglyceride, hsCRP, left ventricular ejection fraction (LVEF) and N-terminal pro-brain natriuretic peptide (NT-proBNP) among the three groups were statistically significant. Given the association between low transthyretin concentrations and heart failure, the heart diseases of the patients were described. The proportion of patients with heart failure in the three groups was differed significantly (*P* = 0.008). Patients with transthyretin concentration ≤ 15 mg/dL were older, had lower BMI, poorer nutritional status (lower albumin and triglyceride levels), were more likely to be in an inflammatory state (higher hsCRP levels), had higher NT-proBNP concentration, lower LVEF, and a higher prevalence of diabetes and heart failure. Furthermore, these patients were more likely to have lower levels of physical activity and higher rates of frailty.

**Table 1:** Baseline characteristics of individuals classified by transthyretin percentile

Characteristic	Overall (*n* = 636)	≤ 5th Percentile (*n* = 40)	> 5th−95th Percentile (*n* = 572)	> 95th Percentile (*n* = 24)	*P* value
Transthyretin concentration, mg/dL	[4.59,51.00]	[4.59,15.00]	(15.00,34.00]	(34.00,51.00]	< 0.001
Age, y	74.70 (69.30,80.10)	78.45 (75.35,81.28)	74.40 (69.20,80.10)	70.85 (68.00,75.95)	< 0.001
Male, No. (%)	335 (52.7)	21 (52.5)	297 (51.9)	17 (70.8)	0.344
BMI, kg/m^2^	25.09 ± 3.48	23.15 ± 4.84	25.24 ± 3.35	24.75 ± 2.72	0.044
Diabetes, No. (%)	227 (35.7)	21 (52.5)	202 (35.3)	4 (16.7)	0.014
HbA1c, %	6.20 (5.80, 6.90)	6.45 (5.80, 8.50)	6.20 (5.80, 6.90)	6.00 (5.85, 6.65)	0.169
Smoking, No. (%)	210 (33.0)	13 (32.5)	186 (32.5)	11 (45.8)	0.483
Alcohol, No. (%)	212 (33.3)	9 (22.5)	189 (33.0)	14 (58.3)	0.026
Physical activity, No. (%)	481 (75.6)	22 (55.0)	439 (76.7)	20 (83.3)	0.028
ALT, U/L	15.00 (12.00,22.00)	17.00 (13.00,29.75)	15.00 (11.00,21.00)	18.00 (13.75,23.00)	0.633
Albumin, g/L	40.0 (38.00,42.00)	35.0 (32.48,38.00)	40.00 (38.00,42.00)	42.00 (39.95,45.00)	< 0.001
Cholesterol, mmol/L	3.74 (3.13, 4.33)	3.59 (2.80, 4.18)	3.73 (3.16, 4.32)	4.33 (3.54, 5.04)	0.633
Triglycerides, mmol/L	1.16 (0.84, 1.57)	0.91 (0.71, 1.20)	1.16 (0.84, 1.58)	1.48 (1.14, 2.70)	< 0.001
Low-density lipoprotein, mmol/L	2.16 (1.70, 2.74)	2.12 (1.56, 2.61)	2.16 (1.71, 2.72)	2.32 (2.01, 3.24)	0.083
Creatinine, umol/L	69.00 (59.00, 86.00)	70.00 (55.58, 88.00)	69.00 (58.50,85.00)	74.50 (60.75,89.50)	0.555
hsCRP, mg/L	1.17 (0.63, 2.78)	12.46 (1.46, 28.25)	1.13 (0.62, 2.39)	0.98 (0.64, 1.91)	< 0.001
Fried, No. (%)					< 0.001
0	126 (19.8)	4 (10.0)	112 (19.6)	10 (41.7)	
I	348 (54.7)	16 (40.0)	319 (55.8)	13 (54.2)	
II	162 (25.5)	20 (50.0)	141 (24.7)	1 (4.2)	
NT-proBNP, pg/mL	168.20 (77.43, 557.85)	531.05 (158.70,1508.50)	162.30 (75.81,509.55)	156.00 (65.68, 277.47)	< 0.001
Hypertension, No. (%)	471 (74.1)	27 (67.5)	426 (74.5)	18 (75.0)	0.533
Angina pectoris, No. (%)	279 (43.9)	14 (35.0)	252 (44.1)	13 (54.2)	0.192
Myocardial infarction, No. (%)	80 (12.6)	6 (15.0)	71 (12.4)	3 (12.5)	0.829
Atrial fibrillation /atrial flutter, No. (%)	132 (20.8)	10 (25.0)	117 (20.5)	5 (20.8)	0.713
Heart failure, No. (%)	116 (18.2)	17 (42.5)	97 (17.0)	2 (8.3)	0.008
LVEF(%)	64.00 (60.00, 65.00)	60.00 (38.50, 63.00)	64.00 (60.00,65.00)	65.00 (60.00,65.00)	0.001

*Abbreviations* BMI: body mass index; HbA1c: Hemoglobin A1c; ALT: alanine aminotransferase; hsCRP: high-sensitivity C-reactive protein; NT-proBNP: N-terminal pro-brain natriuretic peptide; LVEF: left ventricular ejection fraction

*Transthyretin* I was defined as transthyretin concentration ≤ 15 mg/dL; III was defined as transthyretin concentration > 34 mg/dL. Fried: 0 was defined as robust; I was defined as prefrail; II was defined as frailty

### Three-year mortality and readmission

**Fig. 2:**
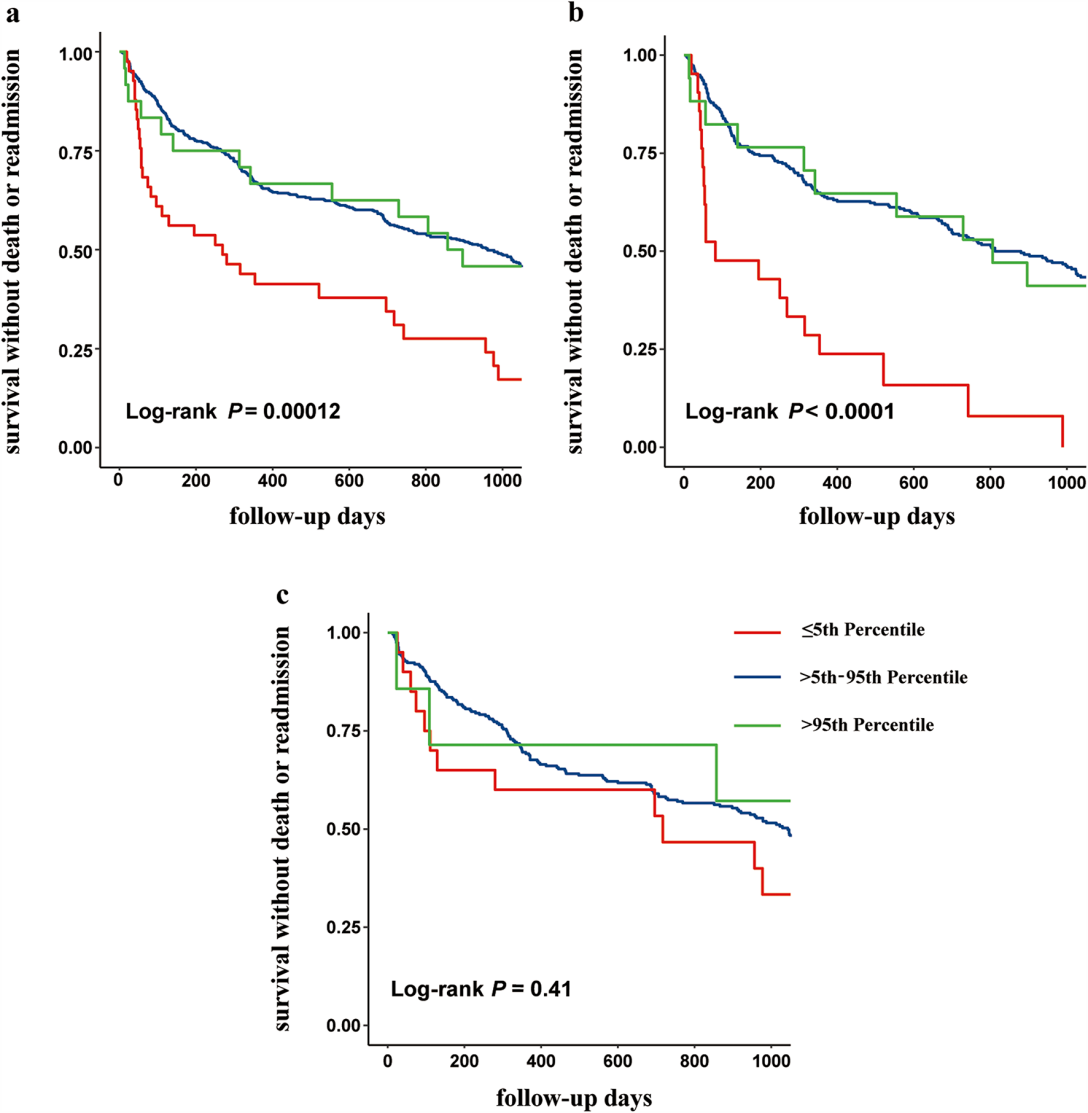
Kaplan-Meier survival curves by transthyretin percentile. **a**: Overall Population; **b**: Male Patients; **c**: Female Patients

During the 3-year follow-up period, 363 (57.0%) patients experienced endpoint events. The incidence of these events varied across transthyretin concentration groups: patients with transthyretin ≤ 15 mg/dL experienced events in 31 cases (77.5%), those with transthyretin concentration between (15, 34] mg/dL experienced events in 318 cases (55.5%), and those with transthyretin > 34 mg/dL experienced events in 14 cases (58.3%). As shown in Fig. 2a, the average survival times differed significantly among these groups (log-rank *P* = 0.00012).

Gender-specific survival analysis revealed that for males, survival times differed significantly among transthyretin concentration groups (log-rank *P* < 0.0001), as depicted in Fig. 2b. In contrast, for females, there was no significant difference in survival times across the transthyretin concentration groups (log-rank *P* = 0.41), as shown in Fig. 2c.

### Relationship between transthyretin concentration and prognosis in elderly inpatients

The study had a median (IQR) follow-up time of 1,099.0 (1,016.3, 1,135.0) days and included 636 elderly hospitalized patients. Of these patients, 363 (57.0%) experienced an endpoint event. Lower transthyretin concentrations is associated with the occurrence of endpoint events.

**Fig. 3:**
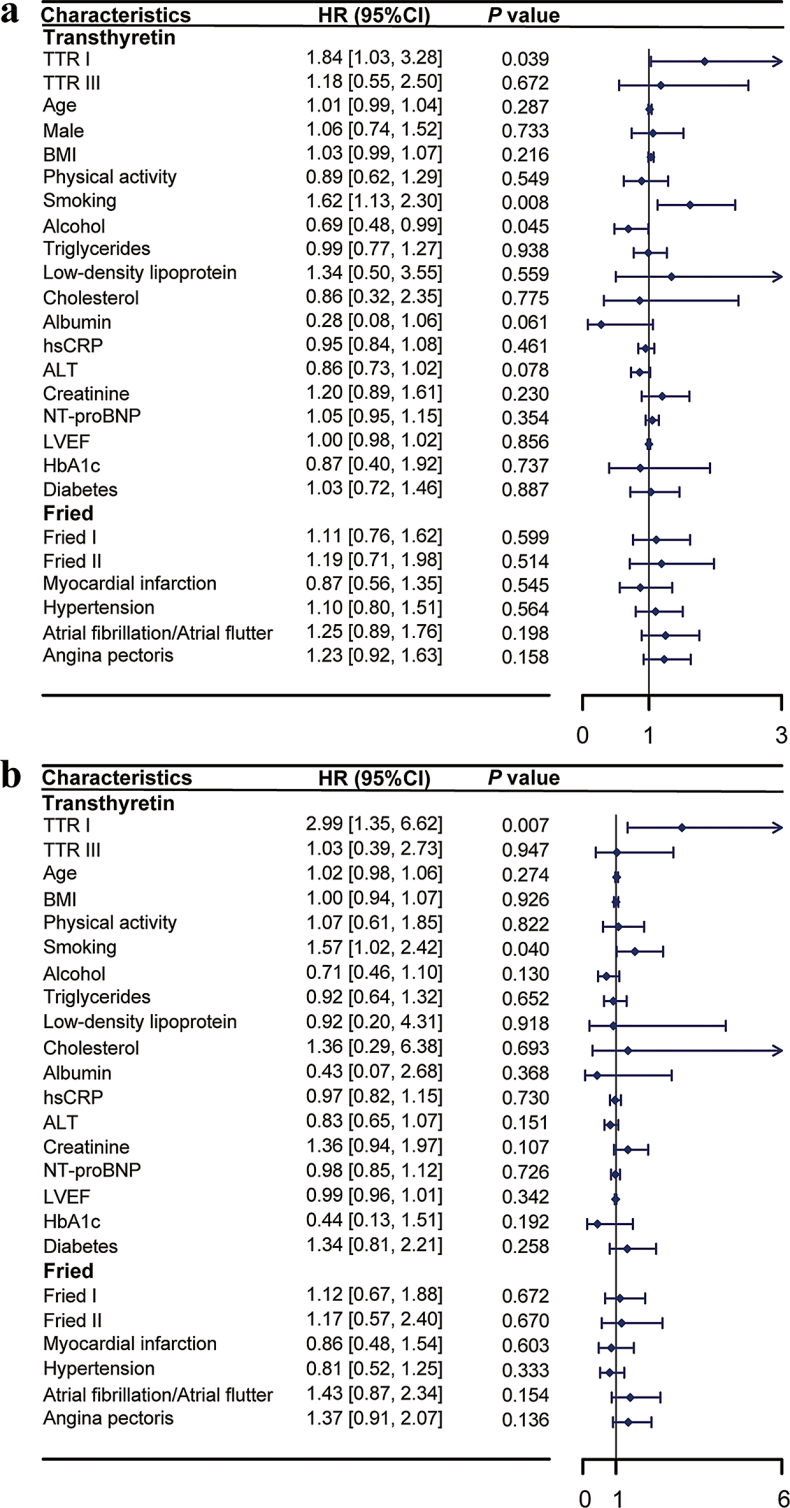
Multivariate Cox regression analysis of transthyretin concentrations and prognosis. **a**: Overall Population. **b**: Male Patients. Transthyretin: I was defined as transthyretin concentration ≤ 15 mg/dL; III was defined as transthyretin concentration > 34 mg/dL. Fried: 0 was defined as robust; I was defined as prefrail; II was defined as frailty

The univariate Cox regression analysis revealed that patients with transthyretin concentrations at or below the 5th percentile had an HR of 2.25 (95% CI: 1.55–3.26) for all-cause mortality and readmission compared to those with transthyretin concentrations between the 5th and 95th percentiles (as detailed in Supplementary Table 2). Figure 3 presents the results of the multivariate Cox regression analyses. Factors associated with the outcomes and related to transthyretin concentration were included in the multivariate Cox regression analysis. Although heart failure was significant in the univariate analysis, it was not included in the multivariate model because NT-proBNP and LVEF are both indicators of heart failure. The results, depicted in Fig. 3a, show that low transthyretin concentration remains an independent risk factor, with an HR of 1.84 (95% CI: 1.03–3.28).

For male patients, univariate analysis revealed an HR of 3.59 (95% CI: 2.22–5.82) for all-cause mortality and readmission in those with transthyretin concentrations at or below the 5th percentile compared to those with concentrations between the 5th and 95th percentiles(see Supplementary Table 3 for details). After adjusting for factors associated with the outcomes and related to transthyretin concentration, the HR for low transthyretin concentration remained significant at 2.99 (95% CI: 1.35–6.62), as shown in Fig. 3b. In contrast, for female patients, the univariate analysis indicated an HR of 1.47 (95% CI: 0.82–2.65), and no significant association with prognosis was observed (see Supplementary Table 4 for details).

**Fig. 4:**
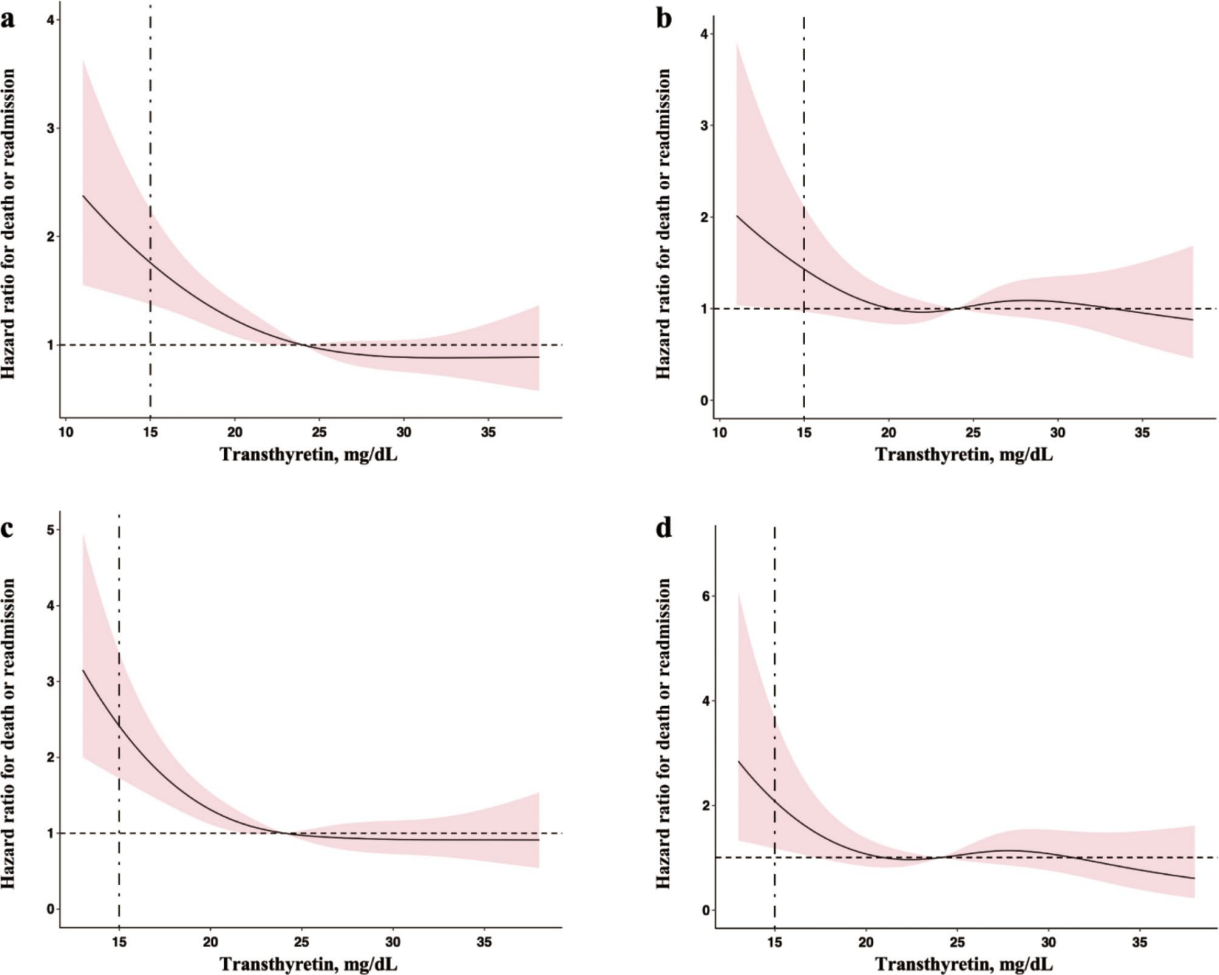
Restricted cubic spline analysis of transthyretin concentration and prognosis. **a**: Relationship between transthyretin concentration and prognosis in the overall population without adjusting for covariates; **b**: Relationship in the overall population adjusted for factors included in the Cox regression model; **c**: Relationship for male patients without adjusting for covariates; **d**: Relationship for male patients adjusted for factors included in the Cox regression model. The vertical dashed line in the panels represents the critical value of transthyretin concentration at 15 mg/dL

Figure 4 provides a visual representation of the relationship between transthyretin concentration and prognosis in elderly inpatients through restricted cubic splines. Panel a shows the unadjusted analysis for the overall population, while panel b displays the results after adjusting for factors included in the Cox regression model. Panel c presents the unadjusted analysis for male patients, and panel d shows the results for males after adjusting for Cox-regression factors. These plots help to illustrate the impact of low transthyretin concentration on poor prognosis more clearly.

## Discussion

Transthyretin, which moves quickly in plasma protein electrophoresis and resides ahead of albumin, hence termed prealbumin, is primarily synthesized in the liver. Compared to albumin synthesized in the liver, prealbumin has a shorter half-life, faster turnover rate, and is less influenced by various factors during measurement, making it more sensitive to changes in patient health status. Prealbumin serves not only as a nutritional marker but also frequently functions as an independent risk factor in certain pathological conditions, aiding in the prediction of patient prognosis. This study focused on the effect of transthyretin concentration on the prognosis of elderly inpatients. We found that low transthyretin concentration is an independent risk factor for mortality and readmission from all causes within three years.

Furthermore, we also found that the elderly inpatients with transthyretin concentration ≤ 15 mg/dL were older, had lower BMI, and had lower levels of albumin, and triglyceride, which may suggest poor nutritional status [18]. In addition, higher hsCRP concentration indicated that patients in this group are more likely to suffer from inflammation [19]. Moreover, higher NT-proBNP concentration and lower LVEF demonstrated a higher prevalence of heart failure in this group. And among these patients, there is a high prevalence of diabetes, physical inactivity and frailty. The underlying reasons for decreased transthyretin concentration may be associated with chronic inflammation, malnutrition, metabolic disorders, and other factors [6]. To ascertain whether low transthyretin is an independent risk factor for adverse clinical outcomes, we performed Cox regression analyses adjusting for the aforementioned variables. The results demonstrated that even after these adjustments, low transthyretin levels remained significantly associated with adverse outcomes.

Consistent with previous findings, low transthyretin concentration is associated with adverse outcomes. A prospective study of peritoneal dialysis patients followed for up to 21 months showed that transthyretin concentrations < 30 mg/dL were associated with increased mortality compared with higher transthyretin concentrations [20]. Similarly, a lower transthyretin concentration at admission in stroke patients is an independent predictor of long-term mortality after mechanical thrombectomy [21]. Additionally, acute heart failure patients with transthyretin concentration ≤ 15 mg/dL at discharge have higher 6-month morbidity and mortality [14].

In our study, we found that low transthyretin concentrations are a risk factor for adverse outcomes in elderly inpatients, with significant differences observed between male and female patients. Specifically, low transthyretin levels were significantly associated with adverse outcomes in men, but no similar association was observed in women. Previous studies have indicated that women generally have lower baseline transthyretin levels than men, and that the destabilization of wild-type transthyretin tetramers may be more common in men, which could explain the stronger association between low transthyretin levels and adverse outcomes in men [16]. Additionally, wild-type transthyretin amyloidosis (ATTRwt) might be a potential mechanism underlying the association between low transthyretin concentrations and increased risk of mortality or readmission. However, as we did not perform specific diagnostic tests for transthyretin amyloidosis, we cannot provide data on patients with low transthyretin levels who may have been diagnosed with amyloidosis during follow-up. We recognize that this potential mechanism has not been fully explored in our study, which is a limitation.

Our research has several limitations. Firstly, some patients meeting the inclusion criteria were excluded due to incomplete transthyretin testing. This exclusion may introduce selection bias and limit the generalizability of our findings to the broader population of elderly inpatients. Second, our study had a relatively small number of patients with transthyretin concentrations that were lower than or equal to the 5th percentile and higher than the 95th percentile, which may have affected the robustness of our results. Lastly, We recognize that this is an observational study and that further research is needed to elucidate the biological pathways through which low transthyretin levels might affect mortality or readmission rates. Future studies with larger sample sizes and less selection bias are needed to validate these findings and to explore the underlying biological mechanisms.

## Conclusion

Low transthyretin concentration is associated with adverse outcomes in elderly inpatients, with a gender-specific difference that is particularly pronounced in men. Clinicians should consider incorporating transthyretin levels into the comprehensive prognostic assessment of elderly inpatients, alongside other clinical and laboratory parameters. Identifying patients at higher risk due to low transthyretin levels, especially male patients, allows for early interventions that may potentially improve clinical outcomes.

## Electronic supplementary material

Below is the link to the electronic supplementary material.

Supplementary Material 1

## Data Availability

All data analysed during this study are included in this published article.

## Abbreviations

IQR: Interquartile Range
hsCRP: High-Sensitivity C-Reactive Protein
BMI: Body Mass Index
ALT: Alanine Aminotransferase
HR: Hazard Risk
CI: Confidence Interval
LVEF: Left Ventricular Ejection Fraction
NT-proBNP: N-Terminal Pro-Brain Natriuretic Peptide
HbA1c: Hemoglobin A1c
