# Supplementary material for: Low transthyretin concentration linked to adverse prognosis in elderly inpatients
Source: BMC Geriatr. 2024 Oct 30;24:893. doi: 10.1186/s12877-024-05467-3 (PMC11523828; doi:10.1186/s12877-024-05467-3)
Supplement: Supplementary file 1 — Supplementary Material 1 [file 12877_2024_5467_MOESM1_ESM.docx]

Supplementary Table 1: Comparison of baseline characteristics between patients with and without transthyretin measurements

| Characteristic | Overall  (n=1068) | Transthyretin Measured  (n=689) | Transthyretin Not Measured  (n=379) | ***P*** value |
| --- | --- | --- | --- | --- |
| Age, y | 74.75 (69.10, 80.80) | 74.70 (69.30, 80.30) | 74.90 (68.90, 81.10) | 0.54 |
| Male,No. (%) | 565 (52.9) | 360 (52.2) | 205 (54.1) | 0.608 |
| BMI, kg/m^2^ | 25.02 (3.58) | 25.06 (3.46) | 24.94 (3.78) | 0.592 |
| Diabetes, No. (%) | 369 (34.6) | 246 (35.7) | 123 (32.5) | 0.317 |
| HbA1c, % | 6.20 (5.80, 7.00) | 6.20 (5.80, 6.90) | 6.30 (5.80, 7.20) | 0.282 |
| Smoking, No. (%) | 354 (33.1) | 227 (32.9) | 127 (33.5) | 0.905 |
| Alcohol, No. (%) | 370 (34.6) | 230 (33.4) | 140 (36.9) | 0.271 |
| Physical activity, No. (%) | 771 (72.2) | 514 (74.6) | 257 (67.8) | 0.022 |
| ALT, U/L | 15.00 (11.00, 21.00) | 15.00 (11.52, 22.00) | 14.00 (11.00, 20.45) | 0.034 |
| Albumin, g/L | 39.00 (37.10, 41.00) | 40.00 (38.00, 41.22) | 39.00 (37.00, 40.55) | <0.001 |
| Cholesterol, mmol/L | 3.77 (3.16, 4.44) | 3.75 (3.14, 4.35) | 3.86 (3.21, 4.60) | 0.086 |
| Triglycerides, mmol/L | 1.14 (0.83, 1.55) | 1.17 (0.84, 1.57) | 1.10 (0.80, 1.46) | 0.103 |
| Low-density lipoprotein, mmol/L | 2.20 (1.73, 2.80) | 2.17 (1.70, 2.74) | 2.30 (1.79, 3.02) | 0.016 |
| Creatinine, umol/L | 70.00 (59.00, 87.00) | 69.00 (58.20, 85.65) | 72.00 (59.00, 89.00) | 0.108 |
| hsCRP, mg/L | 1.22 (0.66, 3.03) | 1.19 (0.63, 2.88) | 2.62 (1.00, 10.57) | 0.001 |
| Fried (%) |  |  |  | 0.039 |
| 0 | 181 (16.9) | 131 (19.0) | 50 (13.2) |  |
| Ⅰ | 598 (56.0) | 371 (53.8) | 227 (59.9) |  |
| Ⅱ | 289 (27.1) | 187 (27.1) | 102 (26.9) |  |
| NT-proBNP, pg/mL | 173.30  (80.52, 575.40) | 170.30  (78.10, 572.85) | 179.10  (92.30, 684.80) | 0.212 |
| LVEF, % | 64.00 (60.00, 65.00) | 64.00 (60.00, 65.00) | 64.00 (60.00, 65.00) | 0.631 |

Abbreviations: BMI: body mass index; HbA1c: Hemoglobin A1c; ALT: alanine aminotransferase; hsCRP: high-sensitivity C-reactive protein; NT-proBNP: N-terminal pro-brain natriuretic peptide; LVEF: left ventricular ejection fraction.

Fried: 0 was defined as robust; Ⅰ was defined as prefrail; Ⅱ was defined as frailty.

Supplementary Table 2: Univariate and multivariate COX regression analysis of prognosis in elderly inpatients

| Variables | | Univariate analysis | | | | Multivariate analysis | | | |
| --- | --- | --- | --- | --- | --- | --- | --- | --- | --- |
|  |  | HR | 95% CI | | ***P*** value | HR | 95% CI | | ***P*** value |
|  |  |  | Lower | Upper |  |  | Lower | Upper |  |
| Transthyretin | Ⅱ | [Reference] | | | | [Reference] | | | |
|  | Ⅰ | 2.25 | 1.55 | 3.26 | <0.001 | 1.84 | 1.03 | 3.28 | 0.039 |
|  | Ⅲ | 0.98 | 0.57 | 1.67 | 0.940 | 1.18 | 0.55 | 2.50 | 0.672 |
| age | | 1.03 | 1.01 | 1.04 | <0.001 | 1.01 | 0.99 | 1.04 | 0.287 |
| Male | | 1.20 | 0.98 | 1.48 | 0.079 | 1.06 | 0.74 | 1.52 | 0.733 |
| BMI | | 1.00 | 0.97 | 1.03 | 0.983 | 1.03 | 0.99 | 1.07 | 0.216 |
| Diabetes | | 1.29 | 1.04 | 1.59 | 0.019 | 1.03 | 0.72 | 1.46 | 0.887 |
| Smoking | | 1.33 | 1.07 | 1.64 | 0.010 | 1.62 | 1.13 | 2.30 | 0.008 |
| Alcohol | | 1.00 | 0.81 | 1.25 | 0.971 | 0.69 | 0.48 | 0.99 | 0.045 |
| Physical activity | | 0.75 | 0.59 | 0.94 | 0.013 | 0.89 | 0.62 | 1.29 | 0.549 |
| ALT | | 0.97 | 0.85 | 1.11 | 0.657 | 0.86 | 0.73 | 1.02 | 0.078 |
| Albumin | | 0.18 | 0.08 | 0.42 | <0.001 | 0.28 | 0.08 | 1.06 | 0.061 |
| Cholesterol | | 0.83 | 0.61 | 1.15 | 0.263 | 0.86 | 0.32 | 2.35 | 0.775 |
| Triglycerides | | 0.89 | 0.76 | 1.05 | 0.167 | 0.99 | 0.77 | 1.27 | 0.938 |
| Low-density lipoprotein | | 0.89 | 0.64 | 1.23 | 0.486 | 1.34 | 0.50 | 3.55 | 0.559 |
| Creatinine | | 1.55 | 1.27 | 1.90 | <0.001 | 1.20 | 0.89 | 1.61 | 0.230 |
| hsCRP | | 1.14 | 1.04 | 1.24 | 0.005 | 0.95 | 0.84 | 1.08 | 0.461 |
| Fried | 0 | [Reference] | | | | [Reference] | | | |
|  | Ⅰ | 1.40 | 1.04 | 1.88 | 0.024 | 1.11 | 0.76 | 1.62 | 0.599 |
|  | Ⅱ | 2.08 | 1.51 | 2.86 | <0.001 | 1.19 | 0.71 | 1.98 | 0.514 |
| NT-proBNP | | 1.14 | 1.08 | 1.20 | <0.001 | 1.05 | 0.95 | 1.15 | 0.354 |
| LVEF | | 0.98 | 0.97 | 0.99 | 0.003 | 1.00 | 0.98 | 1.02 | 0.856 |
| HbA1c | | 1.21 | 0.72 | 2.05 | 0.472 | 0.87 | 0.40 | 1.92 | 0.737 |
| Hypertension | | 1.20 | 0.94 | 1.52 | 0.143 | 1.10 | 0.80 | 1.51 | 0.564 |
| Angina pectoris | | 1.12 | 0.91 | 1.38 | 0.281 | 1.23 | 0.92 | 1.63 | 0.158 |
| Myocardial infarction | | 1.18 | 0.87 | 1.61 | 0.295 | 0.87 | 0.56 | 1.35 | 0.545 |
| Atrial fibrillation /Atrial flutter | | 1.47 | 1.16 | 1.86 | 0.002 | 1.25 | 0.89 | 1.76 | 0.198 |

Abbreviations: BMI: body mass index; HbA1c: Hemoglobin A1c; ALT: alanine aminotransferase; hsCRP: high-sensitivity C-reactive protein; NT-proBNP: N-terminal pro-brain natriuretic peptide; LVEF: left ventricular ejection fraction.

Transthyretin: Ⅰ was defined as transthyretin concentration ≤15mg/dL; Ⅲ was defined as transthyretin concentration > 34mg/dL. Fried: 0 was defined as robust; Ⅰ was defined as prefrail; Ⅱ was defined as frailty.

Supplementary Table 3: Univariate and multivariate COX regression analysis of prognosis in elderly male patients

| Variables | | Univariate analysis | | | | Multivariate analysis | | | |
| --- | --- | --- | --- | --- | --- | --- | --- | --- | --- |
|  |  | HR | 95% CI | | ***P*** value | HR | 95% CI | | ***P*** value |
|  |  |  | Lower | Upper |  |  | Lower | Upper |  |
| Transthyretin | Ⅱ | [Reference] | | | | [Reference] | | | |
|  | Ⅰ | 3.59 | 2.22 | 5.82 | <0.001 | 2.99 | 1.35 | 6.62 | 0.007 |
|  | Ⅲ | 1.08 | 0.59 | 1.98 | 0.811 | 1.03 | 0.39 | 2.73 | 0.947 |
| age | | 1.03 | 1.01 | 1.05 | 0.002 | 1.02 | 0.98 | 1.06 | 0.274 |
| BMI | | 0.96 | 0.92 | 1.00 | 0.038 | 1.00 | 0.94 | 1.07 | 0.926 |
| Diabetes | | 1.33 | 1.00 | 1.77 | 0.047 | 1.34 | 0.81 | 2.21 | 0.258 |
| Smoking | | 1.24 | 0.93 | 1.65 | 0.142 | 1.57 | 1.02 | 2.42 | 0.040 |
| Alcohol | | 0.93 | 0.70 | 1.23 | 0.610 | 0.71 | 0.46 | 1.10 | 0.130 |
| Physical activity | | 0.76 | 0.55 | 1.05 | 0.095 | 1.07 | 0.61 | 1.85 | 0.822 |
| ALT | | 0.89 | 0.74 | 1.08 | 0.234 | 0.83 | 0.65 | 1.07 | 0.151 |
| Albumin | | 0.13 | 0.04 | 0.40 | <0.001 | 0.43 | 0.07 | 2.68 | 0.368 |
| Cholesterol | | 0.95 | 0.62 | 1.44 | 0.795 | 1.36 | 0.29 | 6.38 | 0.693 |
| Triglycerides | | 0.84 | 0.68 | 1.02 | 0.084 | 0.92 | 0.64 | 1.32 | 0.652 |
| Low-density lipoprotein | | 0.97 | 0.63 | 1.49 | 0.880 | 0.92 | 0.20 | 4.31 | 0.918 |
| Creatinine | | 1.46 | 1.11 | 1.92 | 0.006 | 1.36 | 0.94 | 1.97 | 0.107 |
| hsCRP | | 1.17 | 1.05 | 1.31 | 0.005 | 0.97 | 0.82 | 1.15 | 0.730 |
| Fried | 0 | [Reference] | | | | [Reference] | | | |
|  | Ⅰ | 1.39 | 0.95 | 2.04 | 0.092 | 1.12 | 0.67 | 1.88 | 0.672 |
|  | Ⅱ | 2.04 | 1.34 | 3.11 | 0.001 | 1.17 | 0.57 | 2.40 | 0.670 |
| NT-proBNP | | 1.12 | 1.04 | 1.20 | 0.001 | 0.98 | 0.85 | 1.12 | 0.726 |
| LVEF | | 0.98 | 0.97 | 1.00 | 0.024 | 0.99 | 0.96 | 1.01 | 0.342 |
| HbA1c | | 0.85 | 0.42 | 1.72 | 0.652 | 0.44 | 0.13 | 1.51 | 0.192 |
| Hypertension | | 0.99 | 0.73 | 1.35 | 0.954 | 0.81 | 0.52 | 1.25 | 0.333 |
| Angina pectoris | | 1.14 | 0.86 | 1.51 | 0.349 | 1.37 | 0.91 | 2.07 | 0.136 |
| Myocardial infarction | | 1.09 | 0.75 | 1.59 | 0.637 | 0.86 | 0.48 | 1.54 | 0.603 |
| Atrial fibrillation /Atrial flutter | | 1.45 | 1.05 | 2.01 | 0.022 | 1.43 | 0.87 | 2.34 | 0.154 |

Abbreviations: BMI: body mass index; HbA1c: Hemoglobin A1c; ALT: alanine aminotransferase; hsCRP: high-sensitivity C-reactive protein; NT-proBNP: N-terminal pro-brain natriuretic peptide; LVEF: left ventricular ejection fraction.

Transthyretin: Ⅰ was defined as transthyretin concentration ≤15mg/dL; Ⅲ was defined as transthyretin concentration > 34mg/dL. Fried: 0 was defined as robust; Ⅰ was defined as prefrail; Ⅱ was defined as frailty

Supplementary Table 4: COX regression analysis of prognosis in elderly female patients

| Variables | | Univariate analysis | | | |
| --- | --- | --- | --- | --- | --- |
|  |  | HR | 95% CI | | ***P*** value |
|  |  |  | Lower | Upper |  |
| Transthyretin | Ⅱ | [Reference] | | | |
|  | Ⅰ | 1.47 | 0.82 | 2.65 | 0.200 |
|  | Ⅲ | 0.68 | 0.22 | 2.13 | 0.506 |
| age | | 1.03 | 1.01 | 1.05 | 0.014 |
| BMI | | 1.04 | 1.00 | 1.09 | 0.056 |
| Diabetes | | 1.21 | 0.87 | 1.66 | 0.253 |
| Smoking | | 1.50 | 0.83 | 2.70 | 0.178 |
| Alcohol | | 0.73 | 0.40 | 1.31 | 0.293 |
| Physical activity | | 0.71 | 0.51 | 0.99 | 0.044 |
| ALT | | 1.02 | 0.84 | 1.23 | 0.846 |
| Albumin | | 0.27 | 0.07 | 0.96 | 0.043 |
| Cholesterol | | 0.81 | 0.49 | 1.34 | 0.414 |
| Triglycerides | | 1.05 | 0.81 | 1.36 | 0.708 |
| Low-density lipoprotein | | 0.85 | 0.52 | 1.39 | 0.521 |
| Creatinine | | 1.67 | 1.15 | 2.43 | 0.007 |
| hsCRP | | 1.08 | 0.94 | 1.25 | 0.286 |
| Fried | 0 | [Reference] | | | |
|  | Ⅰ | 1.46 | 0.92 | 2.32 | 0.104 |
|  | Ⅱ | 2.25 | 1.38 | 3.69 | 0.001 |
| NT-proBNP | | 1.17 | 1.08 | 1.27 | <0.001 |
| LVEF | | 0.98 | 0.96 | 1.00 | 0.089 |
| Hypertension | | 1.57 | 1.06 | 2.31 | 0.023 |
| Angina pectoris | | 1.10 | 0.81 | 1.49 | 0.559 |
| Myocardial infarction | | 1.26 | 0.72 | 2.23 | 0.417 |
| Atrial fibrillation/Atrial flutter | | 1.50 | 1.05 | 2.13 | 0.025 |
| Heart failure | | 1.97 | 1.35 | 2.88 | <0.001 |

Abbreviations: BMI: body mass index; HbA1c: Hemoglobin A1c; ALT: alanine aminotransferase; hsCRP: high-sensitivity C-reactive protein; NT-proBNP: N-terminal pro-brain natriuretic peptide; LVEF: left ventricular ejection fraction.

Transthyretin: Ⅰ was defined as transthyretin concentration ≤15mg/dL; Ⅲ was defined as transthyretin concentration > 34mg/dL. Fried: 0 was defined as robust; Ⅰ was defined as prefrail; Ⅱ was defined as frailty.
